# Aesculapius meets Vulcanus: robotic chest surgery

**DOI:** 10.1093/icvts/ivae066

**Published:** 2024-04-24

**Authors:** Tamás F Molnar

**Affiliations:** Medical Skill and Innovation Center, Medical School, University of Pécs, Pécs, Hungary; Medical Technology Competence Center, Szechenyi Istvan University of Győr, Győr, Hungary

**Keywords:** VATS, Robotic-assisted thoracic surgery, Bias, Skill, Training

All arts, surgery included are a mystic amalgam of spiritual or intellectual substance and material or purely technical elements. The perpetual limbo regarding their interdependence defines the practice and the pedagogy of the respective disciplines. The pathos of the introductory statement transformed into postoperative survival, quality of life and characteristics of surgery offer face values and valid parameters. Once upon a time but not very far away surgery continuously pushed the limits becoming more and more extensive and radical in which medical profession defined as progress. In the new millennium access size and removed tissue volume are to be minimized under the same banner of the progress. Some of the most eminent experts associated with the European Society of Thoracic Surgery explored the membership of this community [1] regarding their opinion (experience and/or expectations) in reference to robotic-assisted thoracic surgery (RATS). Minimally invasive surgery (MITS) fast becoming a central paradigm in the new millennium justifies the effort of the authors. Within the 10% response rate, overseas members formed just below one-third of the represented centres beyond the geographic borders of the European continent, extending from the eastern shores of the Atlantic Ocean to the Ural Mountains. (Jordan is definitely off: see Table 3.) At present Video Assisted Thoracic Surgery (VATS) is the dominating access technique among the answering membership while RATS, the new kid in the block, seemingly is but the object of desire, than the reality within arm reach. Apart from the introduction of the single lung ventilation [2] only the implementation of the surgical staplers [3] is comparable in its significance to the present surgical technology paradigm shift in chest surgery. The main message of the report is an unambiguous positive suspense with regard to the arrival of RATS, at least among the responders. Three out of 4 of the lucky few having access to the RATS work in an academic institution indicating that the budgeteers, ‘who pay the piper’ need further evidence. Extramedical considerations are intermingling with surgical ones.

According to the abstract 71% of the answering surgeons recommended adoption of robotics is the future [1]. It remains unclear, how many of them do so based solely on his/her personal experience. Separation of reality from pure impressions and benevolent wishes increases trustworthiness.

Acceptance and implementation of a certain medical technology such as RATS is an interplay of strictly professional, socioeconomical, budgetary, healthcare and social security system and political factors.

Partial permeation of RATS into the standard surgical procedures for other than strictly medical reasons leads to segregation unintentionally resulting in first- and second-rate operations and consequently patients. Supporting cited statement such as ‘advantages of RATS over VATS’ [4] needs waterproof evidence otherwise it remains a reflection on wishful thinking. The question remains open for the future of the techniques found to be inferior in such a comparison. Statistical differences and clinical significance are not always inseparable.

The emphasis regarding the importance of training is one of the strong points of the article. This complex, lengthy and expensive process always combines hard and soft skills. The paper focuses on the first, while the latter in which decision-making is a crucial element is inexplicably missing from the equation. An undesired outcome of unbalanced training results in a trainee knowing ‘how to do’ with insufficient compass for the ‘what and when’, and most importantly ‘what not to do’. An eminent issue is the technical inoperability (i.e. irresectability due to ‘concrete hilum’ and/or extensive consolidation) which judgement has a strong surgeon and technicality/technological variance. There is an obvious yet silent unpaid debt in reporting (and teaching) with regard to complex cases deemed inoperable by MITS however solvable by open procedure and vice versa. We already see abdominal surgeons in trouble when the need for conversion into open procedure arises. MITS definitely challenges our existing educational protocols [5]. The need for ‘open thoracotomy masterclasses’ to avoid the extinction of this capability is yet under the horizon but approaching.

It is up to the subsequent research to offer a balanced perspective regarding the sensitive issue of operative length of time. It consumes not only the surgeon, but staff and the entire anaesthesia team. Contemporary surgery delights in the luxury of lack of pressure regarding time, but theater occupancy is generally counted in Euro/min. An operator seated lateral or with his/her back to the patient, far from the body in the question and the sterile field while is tempted to pay less than ideal to the clock on the wall. Theater engagement is a pressing issue in many hospitals throughout Europe and beyond. There are many parallels between the psychological challenges and the ergonomy issues of the pilots and surgeons. The seated position of operateur—thus far not the standard pose in thoracic surgery—narrows the gap between the 2 professions. The surgeon definitely lacks in engaging the use of autopilot while the robotic intrathoracic procedure frequently takes its time cast over a long-distance flight.

The pathway is laid down by this pathfinder article [1] aiming at creating a map of the receiving field.

Obviously, RATS represents the cutting edge technology with definitive potential, primarily in oncologic surgery. It is as important to the progress of the art of thoracic surgery as Formula 1’s contribution to the car industry, specifically the motor of improvement [6]. Exploring the technological terra incognita ahead of us must be secured by a strict adherence to the basic rules of surgery, otherwise we find the cart well before the horses. The picture is expected to broaden as increasing number of European participants have access to the RATS. Subsequent, nuanced areas of research will likely follow with a balanced view, hopefully with a sequelae to check which expectations and promises are fulfilled and which failed. High-fidelity prognostication is dependent upon quality of the reporting on trends [7]. The validity of this observation is doubled in surgery in which mistakes and defeats teach more than victories, We definitely need more self-reflections, ‘deep water sondas’ in the sea of the ordinary, day-by-day thoracic surgical practice, even in RATS.

**Conflict of interest:** none declared.
